# 3D Printed Osteoblast–Alginate/Collagen Hydrogels Promote Survival, Proliferation and Mineralization at Low Doses of Strontium Calcium Polyphosphate

**DOI:** 10.3390/pharmaceutics15010011

**Published:** 2022-12-20

**Authors:** Shebin Tharakan, Shams Khondkar, Sally Lee, Serin Ahn, Chris Mathew, Andrei Gresita, Michael Hadjiargyrou, Azhar Ilyas

**Affiliations:** 1 Bio-Nanotechnology and Biomaterials (BNB) Lab, New York Institute of Technology, Old Westbury, NY 11568, USA; 2 College of Osteopathic Medicine, New York Institute of Technology, Old Westbury, NY 11568, USA; 3 Department of Bioengineering, New York Institute of Technology, Old Westbury, NY 11568, USA; 4 Department of Biological and Chemical Sciences, New York Institute of Technology, Old Westbury, NY 11568, USA; 5 Department Electrical and Computer Engineering, New York Institute of Technology, Old Westbury, NY 11568, USA

**Keywords:** tissue engineering, 3D printed scaffold, hydrogel, degradation rate, cytotoxicity

## Abstract

The generation of biomaterials via 3D printing is an emerging biotechnology with novel methods that seeks to enhance bone regeneration. Alginate and collagen are two commonly used biomaterials for bone tissue engineering and have demonstrated biocompatibility. Strontium (Sr) and Calcium phosphate (CaP) are vital elements of bone and their incorporation in composite materials has shown promising results for skeletal repair. In this study, we investigated strontium calcium polyphosphate (SCPP) doped 3D printed alginate/collagen hydrogels loaded with MC3T3-E1 osteoblasts. These cell-laden scaffolds were crosslinked with different concentrations of 1% SCPP to evaluate the effect of strontium ions on cell behavior and the biomaterial properties of the scaffolds. Through scanning electron microscopy and Raman spectroscopy, we showed that the scaffolds had a granular surface topography with the banding pattern of alginate around 1100 cm^−1^ and of collagen around 1430 cm^−1^. Our results revealed that 2 mg/mL of SCPP induced the greatest scaffold degradation after 7 days and least amount of swelling after 24 h. Exposure of osteoblasts to SCPP induced severe cytotoxic effects after 1 mg/mL. pH analysis demonstrated acidity in the presence of SCPP at a pH between 2 and 4 at 0.1, 0.3, 0.5, and 1 mg/mL, which can be buffered with cell culture medium. However, when the SCPP was added to the scaffolds, the overall pH increased indicating intrinsic activity of the scaffold to buffer the SCPP. Moreover, cell viability was observed for up to 21 days in scaffolds with early mineralization at 0.3, 0.5, and 1 mg/mL of SCPP. Overall, low doses of SCPP proved to be a potential additive in biomaterial approaches for bone tissue engineering; however, the cytotoxic effects due to its pH must be monitored closely.

## 1. Introduction

Bone fractures affect millions of people annually due to trauma or diseases such as osteoporosis, which increases the risk of fractures due to decreased bone density. The high incidence of osteoporotic fractures is a major health concern as well as an economic burden; the average cost to treat a fracture nonunion is over $10,000 according to a 2014 study detailing the financial burdens in orthopedic surgery [1]. Generally, there are two types of fracture healing, direct and indirect. The direct, or primary, method is a slow process that can take months to years and occurs without callus formation [2,3]. However the absence of callus formation is beneficial for patients to restore the biomechanical properties of normal bone [2,4]. The indirect, or secondary, method is the most common and includes endochondral and intramembranous healing through the formation of cartilage, followed by the replacement of calcified areas with bone [2]. Other methods of bone healing include the use of bone grafts, both biological and synthetic. While being considered the “gold standard,” grafting requires additional surgical procedures, increases risk for infections, and raises concern about the cost for hospital stays and treatment [5]. With cases of skeletal fractures and their associated cost continuing to rise, current investigations to find alternatives, including biomaterials with adequate biomechanical properties that offer osteoinductive and osteoconductive properties are essential.

Three-dimensional bioprinting is revolutionizing the tissue engineering and regenerative medicine field because of its capability to deliver structural biomaterials in a precise and controlled manner for biomolecule and cell delivery [6]. Recent advances in 3D bioprinting have elevated the field into the innovative use of 4D bioprinting and automated live printing technology [7,8,9]. Four-dimensional bioprinted constructs are 3D bio-responsive scaffolds with intrinsic capability for remodeling through physiological stimuli to regenerate damaged tissue [9,10,11]. From these advances, elastomers and shape-memory polymers have been applied to bone, cartilage, and muscular defects and are efficacious in regrowing tissue [12]. As such, dynamic tissue constructs are multifaceted polymers that can be 3D printed with a variety of properties for tissue engineering [13]. Biofunctional scaffolds can be 3D printed in various shapes and sizes influencing the effects on cell delivery and growth within the scaffold. These biomimetic 3D printed scaffolds can promote cell attachment, migration, and proliferation because of their biocompatibility, biodegradability, and in some cases their material properties [14]. Commonly used biomaterials such as polylactic acid, poly-L-lactic acid, and polycaprolactone have been used to create synthetic scaffolding for bone regeneration applications. The dynamic 3D environment provided by these scaffolds allows for robust cell growth, powerful tensile-strength, and biodegradability [15,16]. Depending on the composition of the bioink used, an ideal scaffold should have adequate rigidity to withstand external and internal (blood) pressures and serve effectively for tissue regeneration [17]. A recent study by Noroozi et al. utilized triply periodic minimal surface (TPMS) structures with polylactic acid to create complex geometric bone scaffolding and found the scaffold stiffness related to porosity [18]. Alternatively, biological materials have been shown to be biodegradable and biocompatible for tissue engineering. Biopolymers such as nanocellulose, gelatin, hyaluronic acid, and alginate are utilized in a variety of applications and have been used for both tissue engineering and cosmetics [19,20].

Alginate, a polysaccharide from brown algae, has been used as a hydrogel in tissue engineering because of its cost effectiveness, wide-range of compatibility, and its structural similarity to other proteins already present in the extracellular matrix (ECM) [21]. The polymerization of alginate is rapid, often taking seconds to minutes when exposed to divalent cations, allowing for various ion doping. Unfortunately, the structure of alginate lacks peptide binding sites. Often times, alginate is conjugated to an ECM-based protein or an Arginine-Glycine-Aspartate (RGD) peptide sequence to facilitate cell adhesion [22]. Collagen, a naturally occurring ECM protein, can be combined with alginate to provide cell binding sites, as well as its own benefits such as low immunogenicity and promotion of bone regeneration [23]. While biomaterial cost is a concern, collagen is an essential matrix protein to facilitate cell adhesion and migration in lieu of alginate-conjugated RGD peptide sequences. The usage of collagen matrices has also been shown to guide osteoblast behavior in various hydrogels [24,25].

Calcium phosphate (CaP) offers osteoinductive properties that can be utilized through noninvasive surgeries and limit the possible risk of infection [26]. CaP has been used as a surface coating and incorporated into scaffolds, demonstrating positive effects on inducing osteogenesis and angiogenesis. However, the use of CaP alone exhibits short-lived benefits, while having minimal effects on osteogenesis and possessing degradative properties [26,27]. Thus, we hypothesize that combining the effects of CaP with a trace element, strontium (Sr), to form Strontium Calcium Polyphosphate (SCPP) will minimize the degradative behavior, speed up the healing process, and strengthen the formation of new bone. Lastly, SCPP is a mixture of calcium (Ca) and Sr that has been shown to be favorable for osteoblast growth [28,29,30]. In this study, we generated 3D bioprinted MC3T3-E1 pre-osteoblasts-alginate/collagen scaffolds that were exposed to the liquid state of 1% SCPP as a crosslinking agent, and evaluated the biocompatibility and bio-responsiveness of the SCPP as a supplemental agent. While prior studies utilized SCPP as a ceramic structure for osteoblast growth, we demonstrate the use of liquid SCPP to polymerize alginate/collagen hydrogels to facilitate osteoblast growth and mineralization. The structural characterization of the scaffolds exposed to SCPP was conducted through the use of scanning electron microscopy (SEM), Raman spectroscopy, scaffold breakdown, and swelling. The cellular characterization was conducted through viability assays such as MTS and LIVE/DEAD, and mineralization through alizarin red.

## 2. Materials and Methods

### 2.1. SCPP Synthesis

**Figure 1:**
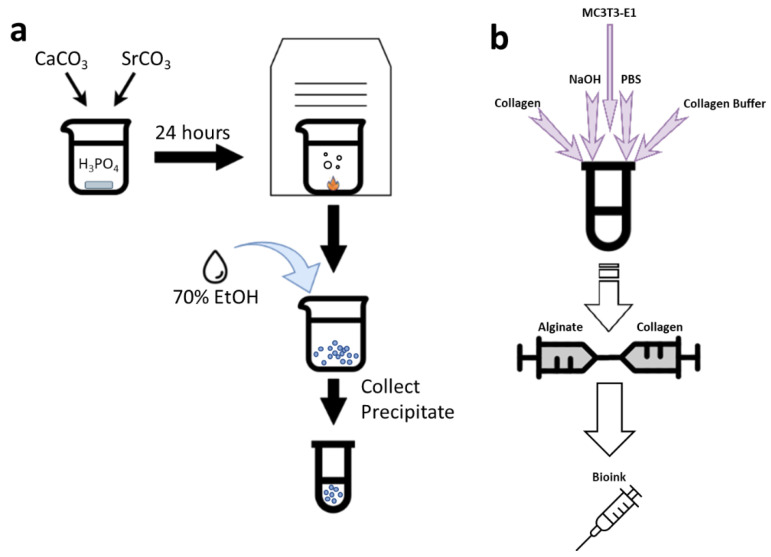
(**a**) Synthesis and collection of 1% Strontium Calcium Polyphosphate (SCPP). Briefly, CaCO_3_ and SrCO_3_ were mixed in H_3_PO_4_ and heated for 24 h and washed with 100% EtOH afterwards. (**b**) Reconstitution and neutralization of type 1 collagen mixed with alginate to form the bioink. The collagen was reconstituted with slightly alkaline pH with the addition of PBS, NaOH, and collagen buffer. After collagen reconstitution, MC3T3-E1 pre-osteoblasts were added to facilitate attachment with the collagen prior to mixing with alginate.

Calcium carbonate (CaCO_3_) (Sigma, St. Louis, MO, USA) and strontium carbonate (SrCO_3_) (Sigma, St. Louis, MO, USA) were mixed at a Ca/Sr ratio of 67.113. The mixture was slowly added to 52 mL of 15% phosphoric acid (H_3_PO_4_) (Sigma, St. Louis, MO, USA) and left overnight while stirring until total dissolution. The solution was then evaporated in a vacuum oven and the 1% SCPP precipitates were collected (Figure 1a). The crystalline precipitates were washed with 100% ethanol to increase the pH. The crystals were then ground to a fine powder and stored in a dry environment at room temperature. Prior to use, the crystals were sterilized under UV light for 30 min and then placed into solutions of deionized water to generate various concentrations (0.1, 0.3, 0.5, 1, 3, 5, and 10 mg/mL). The diluted SCPP solutions were checked for their pH values with an Accumet XL150 benchtop pH sensor (ThermoFisher, Waltham, MA, USA). The capability of α-MEM to buffer SCPP as a semi-physiological environment was evaluated by diluting the SCPP to the desired concentrations in α-MEM. Afterwards, the SCPP-α-MEM solution pH was checked with the benchtop pH sensor.

### 2.2. Cell Culture

MC3T3-E1 pre-osteoblasts (ATCC, Manassas, VA, USA) were cultured in α-MEM (ThermoFisher, Waltham, MA) at 37 °C with 5% CO_2_ until passage 8 and then harvested for bioprinting. Briefly, 0.25% Trypsin-EDTA (ThermoFisher, Waltham, MA, USA) was added to the cell monolayer and then the detached cells were centrifuged to form a cell pellet. The pellet was suspended in a small volume of α-MEM and the cells were counted using a disposable hemocytometer (ThermoFisher, Waltham, MA, USA). Then, the cell solutions with appropriate volume for bioprinting were prepared. Cells were printed at a density of 4 × 10^5^ per scaffold.

### 2.3. SCPP Cytotoxicity

Cells were seeded in a 96-well plate at 2 × 10^4^ cells/well for 1, 3, and 7 days. SCPP was added to the cells at concentrations of 0.1, 0.3, 0.5, 1, 3, 5, 10 mg/mL. Additionally, 100 mM CaCl_2_ (Sigma, St. Louis, MO, USA) was added as a control. Prior to the addition of the MTS reagent (Promega, Madison, WI, USA), the media was removed, and the cells were washed with PBS (ThermoFisher, Waltham, MA, USA) twice. Afterwards, the MTS reagent was added to the wells to quantify cell viability according to the manufacturer’s protocol. After 4 h, the plate was read optically at 490 nm to obtain absorbance values. 

### 2.4. Cell Staining

Cells were seeded in a 24-well plate at 2 × 10^4^ cells/well according to the same protocol described above. The cells were washed with PBS twice to remove any debris, and fixed with 70% ethanol for 20 min and then stained with 1% methylene blue (Sigma, St. Louis, MO, USA) for 5 min. Excess methylene blue was removed by adding deionized water (3 times) to the wells. The stained cells were then imaged under light microscopy (Zeiss Axiovert) (Zeiss, Dublin, CA, USA).

### 2.5. Bioink Synthesis

A 5% Alginate (BICO, Boston, MA, USA) solution was mixed with 5 mg/mL type 1 rat tail collagen (BICO, Boston, MA, USA) to synthesize a composite bioink of 2.5% alginate and 0.04% collagen. Briefly, the collagen was reconstituted with sodium hydroxide (NaOH) (Sigma, St. Louis, MO, USA), PBS, and Collagen Buffer (BICO, Boston, MA, USA) to 0.08% with a pH of 7.0, while on ice, and was mixed in equal parts with 5% Alginate. 2 × 10^6^ cells were suspended in α-MEM and added to the collagen preparation as 10% of the total bioink volume. This cell–collagen solution was mixed with the alginate and homogenized to ensure distribution of the cells (Figure 1b). The bioink was kept on ice to prevent premature collagen cross-linking, then placed into a printer cartridge for extrusion. 

### 2.6. 3D Printing

An extrusion-based printer, the BIO X 3D bioprinter (BICO, Boston, MA, USA) was utilized for 3D printing of porous scaffolds. Prior to each print cycle, UV sterilization was conducted three times with the chamber fan on, and the print bed was wiped with 70% ethanol. The cartridge containing the bioink was loaded onto the print-head and was bed leveled using a surface probe (BICO, Boston, MA, USA). Afterwards, the surface probe was disengaged, and the printing cartridge was manually calibrated to the center of the bottom left well of a 6-well plate (ThermoFisher, Waltham, MA, USA). Once calibrated, the bioink was printed with the following optimized settings: 20 mm × 20 mm × 1 mm model, 20 mm/s print speed, 60 kPa, and 10% infill. A 22G conical tip was used to extrude the bioink into 6-well plates. Scaffolds were immersed in concentrations of 0.1, 0.3, 0.5, and 1 mg/mL of SCPP or 100 mM CaCl_2_ (Control) for 15 min post-printing for crosslinking the hydrogel. Afterwards, the SCPP was removed, and the scaffolds were immersed in α-MEM and incubated at 37 °C with 5% CO_2_.

### 2.7. Scaffold Swelling, Degradation, and pH

Scaffolds (n = 3 per group) without cells were printed and after crosslinking, were dried for 15 min at 50 °C to remove excess crosslinking solution. The scaffolds were then immersed in α-MEM for 24 h. Following α-MEM removal the scaffolds were dried for 15 min at 50 °C to remove excess fluid. The scaffolds were then placed on a scale and the weights were recorded to calculate the swelling ratio using the following equation:

S_s_ = (W_f_ − W_i_)/W_f_ (1)

where S_s_ is the scaffold swelling, W_f_ is the final weight, and W_i_ is the initial weight. Degradation of the printed scaffolds was conducted to a similar procedure; however, they were immersed in PBS without Ca^2+^ and Mg^2+^ for 1, 3, and 7 days After each time point, the PBS was removed and the scaffolds were dried at 50 °C for 15 min to evaporate any excess liquid. The degraded scaffolds were placed on a scale and the weights were recorded. The degradation percentage was calculated using the following equation:

S_d_ = 100(W_f_ − W_i_)/W_f_ (2)

where S_d_ is the degradation (%), W_f_ is the final weight, and W_i_ is the initial weight. The pH of the SCPP–Scaffold solution was evaluated with a benchtop pH sensor to determine if the presence of the scaffold can affect the pH of SCPP. Printed scaffolds (n = 3 per group) were immediately immersed in the SCPP solutions and probed with the Accumet XL150 benchtop pH sensor. Afterwards, the scaffolds were immersed in the SCPP solution for 24 h and then evaluated with the pH sensor.

### 2.8. Raman Spectroscopy and Imaging

Raman spectra analysis of the SCPP was conducted with a DXR2 Raman Spectrometer (ThermoFisher, Waltham, MA). SCPP crystals were transferred onto a disposable microscope slide (VWR, Radnor, PA) and placed in the instrument for analysis. The Raman Spectrometer was operated under the following conditions: A 528 nm laser, 0.1 mW of power, 25 μm slit aperture, and a 10× objective lens. The resulting spectrum was Raman shifted, baseline corrected, and smoothed.

The 3D printed scaffolds were crosslinked, washed with PBS, and were dried at 50 °C for 15 min prior to being placed on a disposable microscope slide. Once the scaffolds were placed in the Raman spectrometer, they were viewed under the Atlµs viewing mode with a 10× objective lens. The laser was focused on the region of interest and an image of the locale was captured. The Raman spectrometer was operated under the following conditions: a 528 nm laser, 0.3 mW of power, 25 μm slit aperture, and a 10× objective lens. Additionally, cell-laden scaffolds were placed under the Raman confocal microscope and imaged under a 10× objective lens.

### 2.9. Scanning Electron Microscopy

The 3D printed scaffolds were crosslinked, washed with PBS, and dried at 45 °C for 10 min to remove excess fluid. Sterile metal pin stubs (Ted Pella, Redding, CA, USA) were grasped with a set of tweezers and mounted on a metal preparation station (Ted Pella, Redding, CA, USA). Black double-sided adhesive carbon tape (Ted Pella, Redding, CA, USA) was applied to the surface of the stub until it fully adhered. The scaffold was laid onto the carbon tape and the stub was inserted into the conducting sample holder. Once the stub was fully inserted, the sample holder was placed into the desktop scanning electron microscope (SEM) (ThermoFisher, Waltham, MA, USA). The SEM was operated on 5 kV with 6.0 Hz. The acquired images were obtained after 3 s of exposure. Images were taken on the scaffold, center, periphery, and connecting filament. Afterwards, the sample holder was ejected, the sample was removed, and the stub was sterilized.

### 2.10. Immunofluorescence

Cell-laden printed scaffolds (n = 3 per group) were washed with PBS and then immersed in the LIVE/DEAD solution (ThermoFisher, Waltham, MA, USA) for 15 min at 37 °C after 3 and 7 days of culture. After 15 min, the LIVE/DEAD solution was removed, and the samples were washed with PBS and stained with DAPI (Sigma, St. Louis, MO, USA) for 15 min at 37 °C. Scaffolds were washed with PBS once more and then visualized using a fluorescent microscope (Zeiss Axiovert) (Zeiss, Dublin, CA, USA) with Lumenera Infinity 3 (Teledyne, ON, Canada). Cell viability measurements were determined through ImageJ. The presence of cells within the cell-laden scaffolds were also confirmed with DAPI. Briefly, cell-laden scaffolds (n = 3 per group) were fixed with 10% formalin (Sigma, St. Louis, MO, USA) for 15 min and then washed with PBS. Afterwards, the scaffolds were placed into Tissue-Tek (Leica, Wetzlar, Germany) at −80 °C for 30 min and then sectioned at 20 µm using a cryomicrotome (Leica, Wetzlar, Germany). The cryosections were then stained with DAPI for 5 min at 37 °C and visualized with the fluorescent microscope.

### 2.11. Scaffold Cell Viability

Cell viability in the cell-laden scaffolds was determined with the MTS assay. Briefly, cell-laden scaffolds (n = 3 per group) were printed, crosslinked, and then washed with PBS. Viability was measured immediately after printing at 3 and 7 days. Scaffolds at 3- and 7-days were incubated at 37 °C. After each time point, the scaffolds were washed with PBS, and then the MTS reagent was added to each scaffold according to the manufacturer’s protocols and the plate was read at 490 nm.

### 2.12. Alizarin Red Staining

Osteoblastic differentiation of MC3T3-E1 cells within scaffolds was achieved using osteogenic differentiation media consisting of 0.01 µM dexamethasone (ThermoFisher, Waltham, MA, USA), 50 µg/mL ascorbic acid (ThermoFisher, Waltham, MA, USA), and 10 mM sodium glycerophosphate (ThermoFisher, Waltham, MA, USA). The cells were cultured in osteogenic differentiation media for 7, 14, 21, and 28 days. The osteogenic differentiation media was replaced every 3 days. Upon termination, scaffolds were fixed with 10% formalin for 15 min and then placed into Tissue-Tek at −80 °C for 30 min. Afterwards, the scaffolds were cryosectioned at 20 µm slices and then stained with 0.01% alizarin red. Sections were then washed once with deionized water to remove excess stain. 

### 2.13. Statistical Analyses

All statistical analysis was conducted in GraphPad Prism 9 with one-way analysis of variance (ANOVA), two-factor analysis of variance (2-way ANOVA), or Student’s *t*-test. All results are expressed as the mean ± standard deviation. A *p* value ≤ 0.05 was considered statistically significant. All experiments were conducted in triplicate. *, **, and *** represent *p* ≤ 0.05, 0.01, and 0.001, respectively.

## 3. Results

### 3.1. Alginate/Collagen Scaffolds Buffer the Acidic SCPP

**Figure 2:**
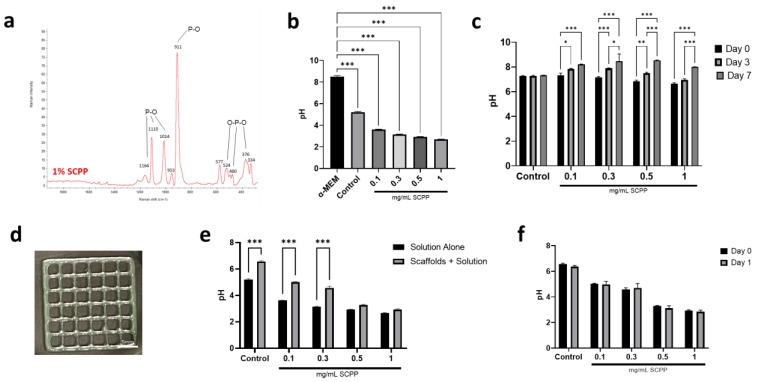
(**a**) Raman spectra of crystalline SCPP after synthesis. (**b**) The pH of α-MEM, CaCl_2_ (control), 0.1, 0.3, 0.5, and 1 mg/mL SCPP solutions immediately after SCPP synthesis and preparation. (**c**) The pH of CaCl_2_ (control), 0.1, 0.3, 0.5, and 1 mg/mL SCPP mixed with α-MEM at day 0, 3, and 7. (**d**) Representative image of a 3D printed alginate/collagen scaffold. (**e**) The comparison between the SCPP solutions with no scaffolds and after adding a scaffold. (**f**) The pH of the scaffold after crosslinking (day 0) and 1 day. * *p* ≤ 0.05, ** *p* ≤ 0.01, *** *p* ≤ 0.001.

Once synthesized, the SCPP was analyzed independently with the Raman spectrometer and with pH analysis. The Raman spectra indicated large peaks at 911, 1014, and 1110 cm^−1^ (Figure 2a). As the SCPP was synthesized from strontium carbonate and calcium carbonate, it retained homologous peaks similar to the original constituents. pH analysis of the SCPP indicated high acidity at concentrations from 0.1 to 1 mg/mL immediately after preparation. The pH levels of 0.1, 0.3, 0.5, and 1 mg/mL SSCP were 3.6, 3.1, 2.9, and 2.6, respectively. The acidity of the SCPP increased as the concentration of SCPP increased (Figure 2b). This is likely due to the acidic solvent (H_3_PO_4_) used in the synthesis of SCPP, causing a lower pH of the crystalline SCPP. It has been previously reported that osteoblasts increased apoptosis and autophagy at acidic pH, while increased differentiation and mineralization is prevalent at basic pH [31,32]. To circumvent premature cell death, we raised the pH of the SCPP by attempting to buffer it with α-MEM. The addition of α-MEM to the SCPP successfully increased the pH of all SCPP concentrations to near 7 for up to 7 days, demonstrating that the acidity can be buffered (Figure 2c). The pH of 0.1, 0.3, 0.5, and 1 mg/mL SCPP solutions after adding α-MEM were 7.3, 7.1, 6.8, 6.6, respectively. After 7 days, the pH for groups containing SCPP rose above 8. This is likely due to the absence of a controlled environment with 5% CO_2_, as 5% CO_2_ maintains the buffering system of α-MEM. As a result, the buffering capability of α-MEM provides a means to minimize the acidity of the SCPP in this semi-physiological environment in vitro, and thus provide appropriate pH for cell survival and growth.

After 3D printing, the scaffolds were gelatinous prior to ion exposure (Figure 2d). Once exposed to SCPP or CaCl_2_ (control), the scaffolds polymerized and adopted a rigid structure. The presence of the carboxylic acids in the alginate polymeric structure allows for the interaction between divalent cations, permitting ionic crosslinking [33,34]. Previous studies involving bioprinted alginate have used CaCl_2_–Alginate inks that were crosslinked prior to 3D printing at low concentrations [35]. Unfortunately, the addition of SCPP to the alginate prior to printing resulted in compact gelation within the printing cartridges that were unable to be extruded; hence, the SCPP and CaCl_2_ were added after printing. The effect of the 3D printed scaffold in modulating pH in the setting of the SCPP was evaluated. The pH of the SCPP alone was compared to the pH of the scaffold directly after adding SCPP. In the presence of the scaffolds, the pH was significantly increased in the control, 0.1, and 0.3 mg/mL of SCPP; however, there was little elevation at 0.5 mg/mL and above. The control, 0.1, and 0.3 mg/mL groups with the scaffold had a 23%, 30.7%, 36.94% difference compared to the SCPP solution alone. Overall, 0.5 and 1 mg/mL groups with scaffolds had a 11.10% and 8.79% difference, respectively, indicating that at higher concentrations the buffering ability of the scaffold is very limited which places cells at risk for greater cell death due to the unfavorable pH shock. However, at lower doses of SCPP, the scaffolds have the intrinsic ability to buffer SCPP to an extent (Figure 2e). The pH of the scaffolds immersed in SCPP was checked after 24 h to determine if the scaffolds are able to buffer the SCPP over a short period of time. No difference was observed in all groups after 1 day although there was a trend towards lower pH than the control with 0.1, 0.5, and 1 mg/mL SCPP scaffold groups. The modulation of pH was marginal, with the lowest percent difference of 0.74% in 0.1 mg/mL SCPP scaffolds. The highest difference was seen in 0.5 mg/mL SCPP scaffolds with 5.02%. While the scaffolds were able to affect the pH of the SCPP directly after addition, the effect is short-lived and is not reflected after 24 h (Figure 2f). Moreover, the synthesis of the bioink involved the combination of collagen, buffer, NaOH, and PBS, and together these components likely created a slightly alkaline scaffold environment that may have contributed to the pH increase observed shortly after the addition of SCPP.

### 3.2. The Use of Scpp Does Not Change the Microscopic Structure or the Raman Spectra of the Scaffolds

**Figure 3:**
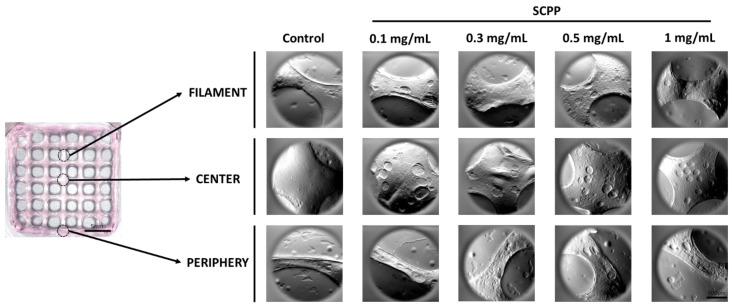
The crosslinked scaffold was segmented into three regions: the center, filament, and periphery. The SEM micrographs for these three regions of interest were captured across the control, 0.1, 0.3, 0.5, and 1 mg/mL groups. The surface morphology appeared to be consistent throughout the groups.

**Figure 4:**
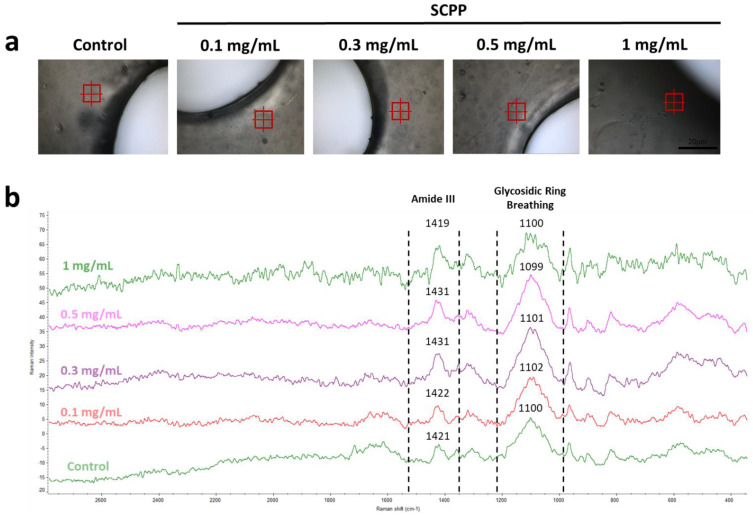
(**a**) Atlµs view of the scaffolds’ topography under the Raman spectrometer. The red crosshairs indicate the region of interest targeted by the 528 nm laser from the Raman microscope. (**b**) The resultant Raman spectra after processing of the control, 0.1, 0.3, 0.5, and 1 mg/mL SCPP groups. Regions of amide III of collagen and the glycosidic ring within alginate were detected.

The microscopic view of the scaffolds indicated a coarse surface with air-filled spaces (Figure 3). The air deposition was likely due to the preparation of the bioink, despite having minimal transfer processes with syringes. The topography across the scaffold from the center, periphery, and filament were granular with some smooth areas. The overall architecture did not vary, indicating that the printing and crosslinking processes did not affect the morphology across groups. Interestingly, collagen fibrillations were not detected with the SEM. This is likely due to the encapsulation of the collagen matrix within the alginate hydrogel. Once the scaffold was polymerized with CaCl_2_ or SCPP and heated, the collagen fibrils likely polymerized and integrated within the scaffold. Raman microscopy of the samples indicates a smooth surface with minor grooves (Figure 4a). Regions of interest were selected from each scaffold and Raman spectra were obtained. The presence of alginate was confirmed with the banding area of the alginate glycosidic ring at 1099–1100 cm^−1^. Furthermore, amide III patterns, reminiscent of collagen, were detected at 1419–1431 cm^−1^. The combination of Raman spectroscopy and SEM indicates that our biomaterial composite maintains the characteristics of alginate but does contain low levels of collagen, as synthesized.

### 3.3. Higher SCPP Concentrations Decrease Scaffold Swelling and Increase Degradation

**Figure 5:**
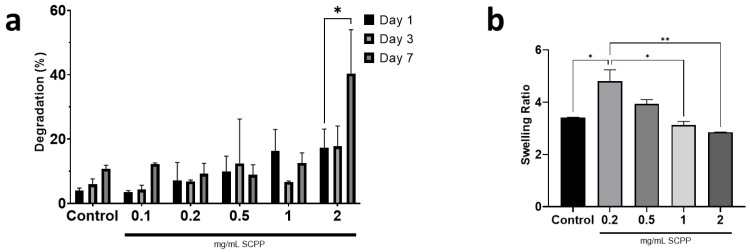
(**a**) Degradation of the scaffolds exposed to SCPP over 7 days. Scaffolds exposed to 2% SCPP had the greatest degradation. (**b**) Swelling of the scaffolds over 24 h indicates scaffolds exposed to 2% SCPP have the lowest swelling ratio. * *p* ≤ 0.05, ** *p* ≤ 0.01.

Degradation and swelling are vital components to the mechanical properties of a hydrogel designed for long-term use [36]. Exposure to higher SCPP concentrations resulted in greater degradation and decreased swelling of the scaffolds. Specifically, the scaffolds with 2% SCPP displayed nearly 40% significant degradation after 7 days, which is a 67.39% increase from day 1 (Figure 5a). The 0.1, 0.2, 0.5, and 1 mg/mL SCPP scaffolds had a comparable degradation percent after 7 days with 12.25%, 9.22%, 8.89%, and 12.58%, respectively. Interestingly, 2% SCPP also yielded the lowest significant swelling ratio of 2.85, compared to the other groups (Figure 5b).

The highest swelling was seen in scaffolds with 0.2 mg/mL of SCPP, with a ratio of 4.82. The decreased swelling in scaffolds with higher SCPP concentrations may be attributed to the increased degradation. The increased degradation in these scaffolds generates fragments that rapidly disintegrate, and thus are unable to swell as efficiently as the intact scaffold. Additionally, lower SCPP concentrations indicated greater swelling compared to CaCl_2_, potentially due to a lower strontium ion content. Strontium ions are chemically larger than calcium ions due to their bigger ionic radius [37] and we hypothesize that due to the larger ionic size of strontium, there is less void space for fluid to enter within the scaffold, resulting in decreased swelling (especially with greater concentrations). The 1% SCPP precipitate is a composite mixture of Ca and Sr, and it is likely that there was a minimal amount of Sr ions present in the solution that led to decreased swelling. Larger molecules may potentially cause molecular distention in the alginate complex that led to the increased degradation over time, as observed with our scaffolds. As bone fractures heal within 4 to 8 weeks [38], scaffolds need slow degradation kinetics to ensure that effective tissue regeneration is achieved before they degrade. The rapid degradation of our alginate/collagen scaffolds make them ideal for short term use only.

### 3.4. Osteoblast Proliferation Is Not Affected in 1 mg/mL SCPP-Doped Scaffolds

**Figure 6:**
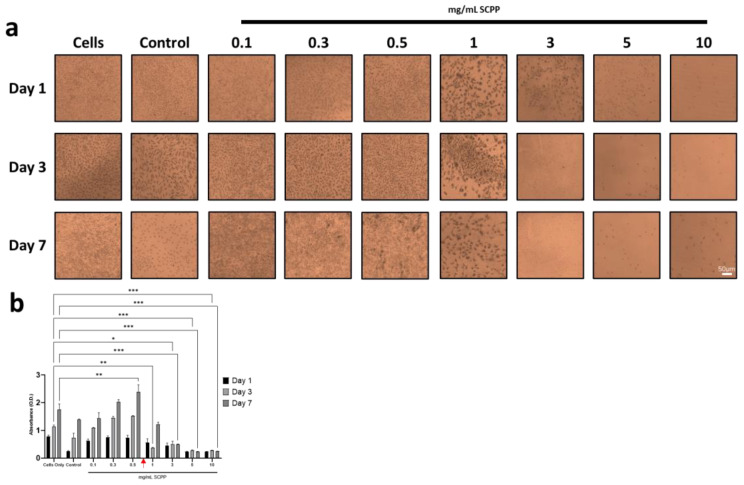
(**a**) Methylene blue staining of cells exposed to 100 mM CaCl_2_ (control) or SCPP from 0.1 to 10 mg/mL across 7 days. Cell death is apparent at 3 mg/mL with blunted cell growth at 1 mg/mL, marking the point of initial cytotoxicity. (**b**) MTS Assay of cells exposed to CaCl_2_ (control) or SCPP across 7 days confirms SCPP cytotoxicity at high doses. Red arrow (in **b**) indicates point of cytotoxicity. * *p* ≤ 0.05, ** *p* ≤ 0.01, *** *p* ≤ 0.001.

To determine the effect of SCPP exposure on viability, cells were exposed to different concentrations of SCPP. Concentrations of 0.1 to 10 mg/mL SCPP indicated dose-dependent cytotoxicity over 7 days. Profound cytotoxic effects were observed following exposure to SCPP concentrations above 1 mg/mL within 3 days (Figure 6a). Concentrations greater than 3 mg/mL of SCPP were observed to be inhibitory for cell growth, as few cells were observed from day 3 to day 7. Interestingly, cells on day 1 that were exposed to 3 mg/mL of SCPP are transiently viable until day 3. This indicates the potential slow acting cytotoxic effect of SCPP. This is accentuated with 5 mg/mL and 10 mg/mL with fewer amounts of cells visible at 1 day and onwards. This is likely due to pH-mediated cell toxicity from the acidity of the SCPP at higher concentrations. Concentrations of 0.1 and 0.3 mg/mL of SCPP did not significantly affect cell proliferation for up to 7 days, although there was an increase over this time period (Figure 6b). However, 0.5 mg/mL of SCPP had a significant effect on cell viability at 7 days, with an absorbance of 2.404 and a 31.54% difference compared to only cells. Proliferation was significantly decreased at 3, 5, and 10 mg/mL. There is a 111.49%, 151.21%, and 149.98% decrease in 3, 5, and 10 mg/mL SCPP groups, respectively, when compared to unexposed cells at day 7.

**Figure 7:**
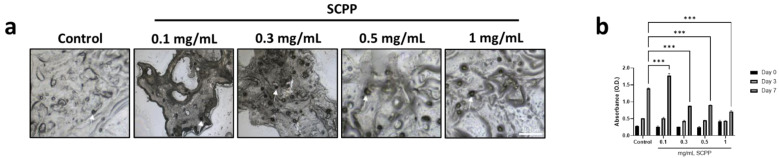
(**a**) Raman microscopy confocal images of scaffolds printed with cells. Cells are dispersed throughout the scaffolds homogenously. White arrows indicate cells embedded on the scaffold surface. (**b**) Cell proliferation of 3D printed cell-based scaffolds over 7 days. *** *p* ≤ 0.001.

Cell-laden scaffolds demonstrated a homogenous distribution of cells throughout, and at all, SCPP concentrations (Figure 7a). Cells were not clustered in one particular region and were spread evenly throughout the scaffold. Further, cell viability was shown to be significantly higher at 0.1 mg/mL of SCPP on day 7, with the absorbance of 1.771 (Figure 7b). Steady cell proliferation was observed in all groups by day 7, however, cell-laden scaffolds with 0.1 mg/mL SCPP were the most biocompatible; there is a 67.62%, 64.53%, and 85.42% greater difference between scaffolds exposed to 0.1 and those at 0.3, 5, and 1 mg/mL SCPP, respectively. Interestingly, persistent cell growth was seen at 1 mg/mL on day 7 (a 51.01% increase from day 1 for 1 mg/mL) indicates that proliferation is attainable in the alginate/collagen hydrogel even when exposed to a near-cytotoxic concentration of SCPP. This may be due to the pH-protective effects of the hydrogel, resulting in a biocompatible environment.

**Figure 8:**
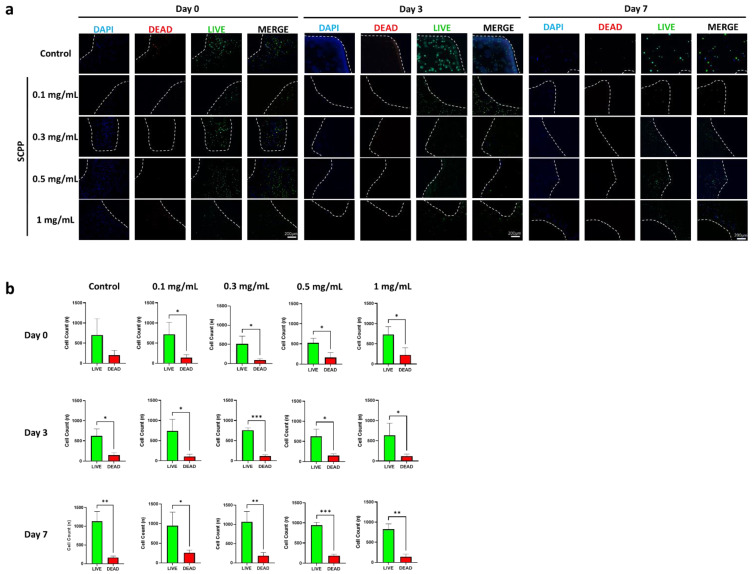
(**a**) LIVE/DEAD and DAPI staining of cell-laden scaffolds after printing, 3, and 7 days. Cells are present throughout the scaffold for up to 7 days. Dashed lines represent the borders of the scaffolds. (**b**) Quantification of LIVE/DEAD fluorescence over 7 days indicates cell growth. The greatest number of dead cells were present on day 0, after printing. * *p* ≤ 0.05, ** *p* ≤ 0.01, *** *p* ≤ 0.001.

LIVE/DEAD and DAPI staining of scaffolds demonstrated living cells with few dead cells on day 0 (after printing). Day 0 had the greatest amount of fluorescent dead cells likely due to printing pressure and the pH shock from the SCPP (Figure 8a). All groups had visible fluorescence of viable cells relative to dead cells from day 3 to 7. Quantitative measurements of cell counts showed a significantly greater number of living than dead cells across 7 days. There was a 1.62, 1.32, 2.07, 1.79, and 1.13-fold increase in live cells in the control, 0.1, 0.3, 0.5, and 1 mg/mL SCPP groups, respectively, between day 0 and day 7.

**Figure 9:**
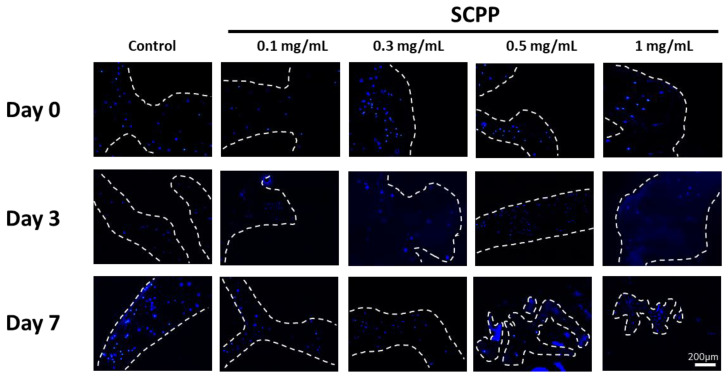
Cryosections of cell-laden scaffolds after printing (day 0), 3, and 7 days indicate cell survival within the biomaterial scaffold through DAPI staining. The presence of cells until day 7 indicates continuous cell growth within the scaffolds. Dashed lines represent the borders of the scaffolds.

The greatest dead cells were seen in scaffolds exposed to 1 mg/mL SCPP with a 1.14-fold increase compared to the control dead cells at day 0. Cell counts on day 7 indicated growth in all scaffolds and there was a 1.19-fold reduction of dead cells in the control group. Unfortunately, while cell counts indicate growth over 7 days, there are also greater dead cells. Groups with 0.1, 0.3, and 0.5 mg/mL SCPP demonstrated a 1.85, 2.10, and 1.13-fold increase in dead cells at day 7, respectively. While this analysis confirms the scaffold’s biocompatibility, cell death persisted likely due to the acidic environment (Figure 8b). The presence of cells within sections of the scaffolds were confirmed with DAPI for up to 7 days indicating the survival of cells (Figure 9). There was no focal area with cells as they were dispersed throughout 7 days, indicating no preferential migration in our hydrogel. This may be due to the rigid alginate structure encapsulating the cells. Additionally, the low collagen concentration likely played a role in providing a low amount of ECM for migration.

### 3.5. Early Osteoblast Calcification Is Seen in 0.3, 0.5, and 1 mg/mL SCPP Doped Scaffolds

**Figure 10:**
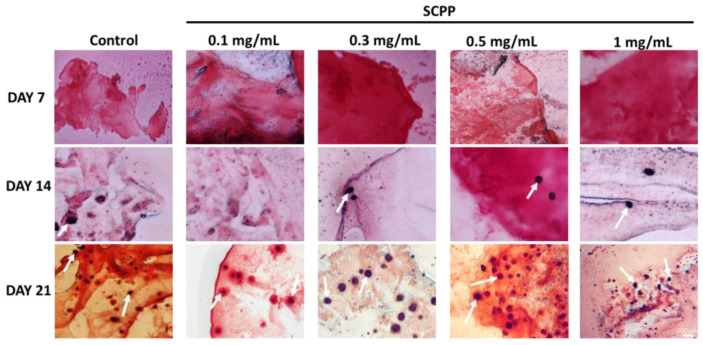
20-micron cryosections stained with Alizarin red for 7, 14, and 21 days. Early nodules are seen on day 14, with the greatest amount of nodule formation at day 21 for each group. White arrows indicate positive alizarin red staining due to osteoblast calcium deposition.

The DAPI stained scaffold cryosections also demonstrated the presence of cells throughout the scaffolds for up to 21 days (Supplementary Figure S1), and thereby providing evidence that the scaffolds are able to sustain long-term cell growth. While sustainable cell growth is an important factor, rapid mineralization and calcification of osteoblasts are also vital components for bone regeneration. Osteogenic induction through differentiation factors facilitates osteoblast growth and osteoid calcification [39]. We observed calcium presence through alizarin red staining in 0.3, 0.5, and 1 mg/mL SCPP doped scaffolds. Interestingly, 0.1 mg/mL SCPP indicated no positive calcium deposition and thus may not be sufficient to stimulate osteoblasts to produce and calcify the matrix. Day 21 demonstrated the greatest calcification across all groups, with the most appearing with equal to or greater than 0.5 mg/mL of SCPP (Figure 10). Thus, 0.5 mg/mL SCPP may be the sufficient amount needed to generate calcium nodules, indicating the potential efficacy in long-term calcification.

## 4. Discussion

Overall, 3D bioprinting is a robust evolving field that provides innovative solutions for regenerative medicine. Three-dimensional printed scaffolds can be seeded with cells or can be directly printed within scaffold polymers. In our study, we directly printed MC3T3-E1 cells within alginate/collagen bioink. Previously, it was shown that high extrusion pressures impose shear stresses on cells that are being bioprinted, leading to increased rates of cell death. In particular, cell printing does not affect viability at pressures less than 5 kPa, while it strongly does at pressures greater than 10 kPa [40]. Our bioink was extruded at a pressure of 60 kPa, due to the high viscosity of alginate, thus requiring greater extrusion pressures. In turn, there was a large amount of dead cells after printing as 60 kPa, likely due to the shear pressure that can damage and lyse cells around the printing nozzle. Interestingly, Taymour et al. report 3D printed hepatocytes in alginate-based bioinks printed at pressures between 50 to 70 kPa [41]. Similar to our results, viability after printing demonstrated large amounts of dead cells. Despite this, long term survival and even proliferation of the surviving cells was not affected by the printing process. The alginate/collagen scaffold was shown to support cell adhesion, survival, proliferation, and mineralization, and thus it is an appropriate biological-based bioink. Previous studies have established alginate as a structural bioink with a lack of integrins for cell adhesion. Common modifications with the arginine–glycine–aspartic acid (RGD) motif or the addition of ECM proteins have been shown to increase cell attachment [42]. In our study, we utilized collagen to facilitate cell adhesion, making up for the lack of integrin binding sites in the alginate [43]. This permitted the cells to attach on a natural ECM protein within the scaffold. The use of ECM proteins is common in tissue engineering. Zhang et al. demonstrated that human umbilical cord mesenchymal stem cells have persistent cell adhesion for 7 days in a similar alginate/collagen hydrogel [44]. Similarly, Sun et al. used MC3T3-E1 osteoblasts in a modified alginate/collagen mold with continued cell growth for up to 5 days [45]. While Sun et al. demonstrated that the cells were able to grow without the addition of collagen, previously, it was shown that human adipose-derived stem cells are able to express their own integrin proteins after a few days to facilitate adhesion onto an alginate hydrogel [46]. Furthermore, the addition of collagen was also shown to increase osteogenic gene expression and is vital in upregulating key markers for bone formation [47].

SCPP is a crystalline material with a co-mixture of calcium and strontium. Previously, it was shown that SCPP is able to support cell proliferation but induces cytotoxicity at high concentrations [28]. This study suggested a concentration of 1% SCPP was necessary to optimize ROS17/2.8 osteoblast proliferation. However, the authors fabricated the SCPP crystals into a CaP scaffold that contained seeded ROS17/2.8 osteoblasts [28]. In contrast, we expose encapsulated MC3T3-E1 cells in alginate/collagen hydrogels with varying concentrations of SCPP in a liquid state. As a result, we demonstrated robust cell growth in a hydrogel environment that provides a shielding effect against cytotoxicity. While the direct cellular cytotoxicity was observed in a non-hydrogel environment, we also demonstrated decreased cell growth at greater SCPP concentrations similar to Qiu et al. [28]. The SCPP utilized in our study had low pH in solution and likely contributed to the cytotoxicity observed, limiting the use of 1% SCPP as a liquid solution compared to a 1% SCPP CaP scaffold. We also demonstrated the cytotoxic effect of 1% SCPP when used at a concentration greater than 1 mg/mL in our experiments, but at lower concentrations, cell proliferation was enhanced for up to 7 days. As such, the dose-dependent cytotoxicity of SCPP allows it to be used at a controlled amount to prevent cell death.

Modulation of the physical components of hydrogels is necessary to fabricate appropriate bioresponsive implants in order to take advantage of their swelling and degradative properties. The swelling of SCPP-doped scaffolds indicates decreased swelling after 24 h, with increasing concentration. Additionally, there is increased degradation at 2 mg/mL SCPP. As bone healing takes place over 4 to 8 weeks, bioresponsive implants need to be biodegradable within this time frame to avoid early degradation [48]. The degradation kinetics observed in our scaffolds are unfavorable as bioresponsive implants, since we see rapid degradation within 1 week. The alginate shells fabricated by Perez et al. indicated similar degradation kinetics in PBS [49]. The 3% alginate had 22.4% degradation within 7 days compared to the 40.39% seen in our 2 mg/mL SCPP group. Furthermore, spectral analysis confirmed the presence of alginate and collagen in our 3D hydrogel. Prior studies demonstrated alginate’s characteristic glycosidic ring around 1100 cm^−1^ and collagen peaks around 820–939 cm^−1^ and 1400 cm^−1^ [50,51]. Imaging of the hydrogel surface indicates a coarse surface with collagen fibrils poorly visualized. Im et al. have shown an alginate–collagen surface coating on polycaprolactone through SEM [52]. Similar to our study, fibril visualization is not seen in the composite mixture. We hypothesize that this may potentially be due to collagen integration within the alginate.

Biopolymers and additive molecules must be able to sustain cell growth so that re-cellularization can restore injured areas. In our study, biocompatibility was observed with sustained cell growth in the scaffolds with CaCl_2_ and SCPP. As a protective feature, the cytotoxic effects of SCPP are blunted when cells are encapsulated inside the hydrogel. Previously, biocompatibility was shown with hydrogel systems to provide a favorable environment for cell growth [53]. The use of 3D cell cultures and scaffolds have been known to facilitate cell division and differentiation to generate cell-laden implantable devices for bone repair. Our alginate/collagen scaffolds confer cell growth without SCPP and are limited with SCPP at concentrations greater than 0.1 mg/mL. In the case of bone regeneration, early mineralization and robust cell division allow for rapid tissue regeneration and the deposition of Ca which is important in the maintenance and development of bone [54,55]. The addition of SCPP demonstrated early calcification at 14 days with the use of 0.3, 0.5, and 1 mg/mL SCPP. Marycz et al. used bone marrow stem cells in alginate hydrogels and determined calcification using Alizarin red staining [56]. Similar to the alizarin red staining in our alginate/collagen hydrogels, the matrix of the alginate hydrogel was able to stain concurrently with the positive cells. This is likely due to the nature of hydrogels being able to soak up liquid, resulting in the non-specific matrix staining seen in our study. As such, the use of SCPP (at the appropriate concentration and formulation) to induce calcium deposition can provide a faster route to increase bone mineralization in a hydrogel environment.

## 5. Conclusions

Our 2.5% alginate and 0.04% type 1 collagen scaffolds doped with SCPP are biodegradable, biocompatible, and osteoconductive. We demonstrated sustained cell growth in the SCPP-doped scaffolds. We also demonstrated the printability of MC3T3-E1 cells mixed directly into our composite bioink. The optimal concentration of 1% SCPP was determined to be between 0.3 and 0.5 mg/mL for cell proliferation, mineralization, and to minimize the cytotoxic effects. Based on our in vitro data, we believe that the composite biopolymer of alginate and collagen is a suitable material for regenerative medicine and bone tissue engineering, given its cytoprotective effects. The use of SCPP, however, is largely limited by its dose-dependent effects. As an additive, it provides rapid cell growth and mineralization at low concentration, but at high concentration it becomes cytotoxic. Future studies should place an emphasis in utilizing divalent cations, particularly Sr, for alginate-based composite bioinks to modulate the scaffold’s degradation rate and evaluate osteoblast responsiveness. We demonstrate the use of liquid SCPP, in contrast to a ceramic CaP structure, as a delivery modality for ionic crosslinking for osteoblast manipulation in 3D hydrogels. Therefore, our study can open new horizons for soluble materials with cationic elements to be used in alginate-based composite bioinks for tissue engineering and drug delivery applications.

## Data Availability

The data presented in this study are available on request from the corresponding authors.

## Supplementary Materials

The following is available online at https://www.mdpi.com/article/10.3390/pharmaceutics15010011/s1, Figure S1: 20-micron cryosections stained with DAPI for 7, 14, and 21 days. Cells are able to live.
