# Supplementary material for: 3D Printed Osteoblast–Alginate/Collagen Hydrogels Promote Survival, Proliferation and Mineralization at Low Doses of Strontium Calcium Polyphosphate
Source: Pharmaceutics. 2022 Dec 20;15(1):11. doi: 10.3390/pharmaceutics15010011 (PMC9865428; doi:10.3390/pharmaceutics15010011)
Supplement: Supplementary file 1 [file pharmaceutics-15-00011-s001.zip › pharmaceutics-2098935-supplementary.pdf]

Supplementary Materials

3D Printed Osteoblast–Alginate/Collagen Hydrogels Promote Survival, Proliferation and Mineralization at Low Doses of Strontium Calcium Polyphosphate

Shebin Tharakan, Shams Khondkar, Sally Lee, Serin Ahn, Chris Mathew, Andrei Gresita, Michael Hadjiargyrou and Azhar Ilyas

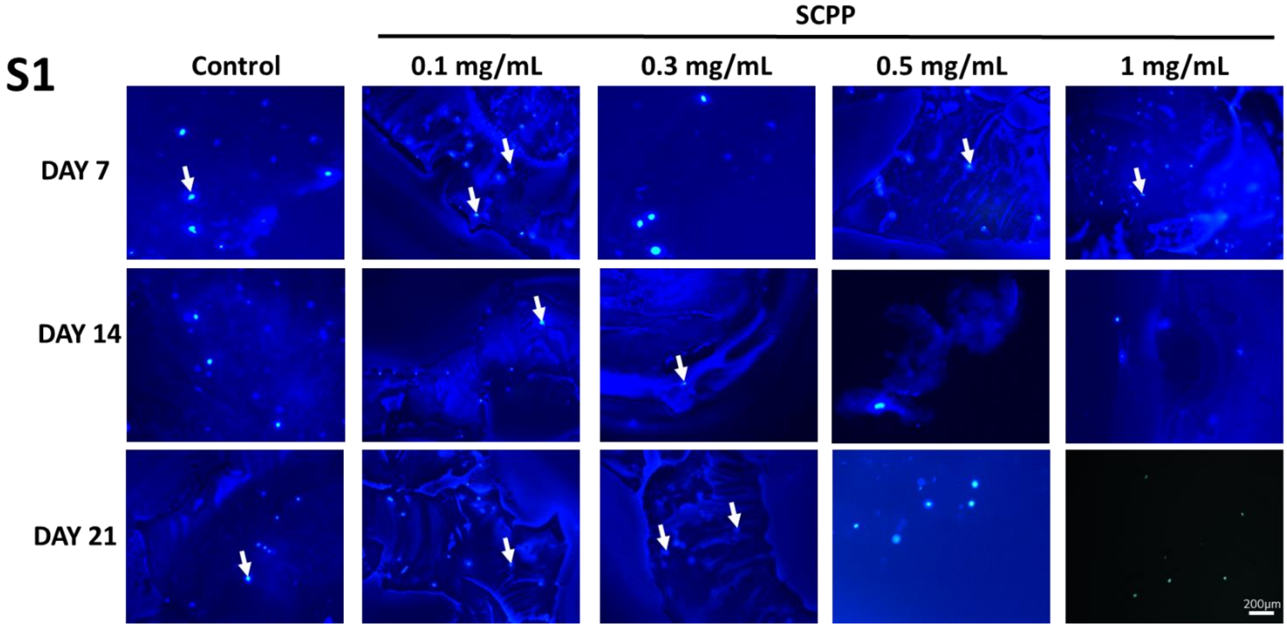

Figure S1. 20-micron cryosections stained with DAPI for 7, 14, and 21 days. Cells are able to live.
